# Novel Insight Into the Epigenetic and Post-transcriptional Control of Cardiac Gene Expression by Thyroid Hormone

**DOI:** 10.3389/fendo.2019.00601

**Published:** 2019-08-29

**Authors:** Francesca Forini, Giuseppina Nicolini, Letizia Pitto, Giorgio Iervasi

**Affiliations:** Consiglio Nazionale delle Ricerche, Institute of Clinical Physiology, Pisa, Italy

**Keywords:** hypothyroidism, low T3 state, cardiac disease, epigenetic regulators, microRNAs, long non-coding RNAs, T3 replacement, combination therapy

## Abstract

Thyroid hormone (TH) signaling is critically involved in the regulation of cardiovascular physiology. Even mild reductions of myocardial TH levels, as occur in hypothyroidism or low T3 state conditions, are thought to play a role in the progression of cardiac disorders. Due to recent advances in molecular mechanisms underlying TH action, it is now accepted that TH-dependent modulation of gene expression is achieved at multiple transcriptional and post-transcriptional levels and involves the cooperation of many processes. Among them, the epigenetic remodeling of chromatin structure and the interplay with non-coding RNA have emerged as novel TH-dependent pathways that add further degrees of complexity and broaden the network of genes controlled by TH signaling. Increasing experimental and clinical findings indicate that aberrant function of these regulatory mechanisms promotes the evolution of cardiac disorders such as post-ischemic injury, pathological hypertrophy, and heart failure, which may be reversed by the correction of the underlying TH dyshomeostasis. To encourage the clinical implementation of a TH replacement strategy in cardiac disease, here we discuss the crucial effect of epigenetic modifications and control of non-coding RNA in TH-dependent regulation of biological processes relevant for cardiac disease evolution.

## Introduction

Remodeling of cardiac tissue architecture is the common denominator of a variety of cardiovascular pathologies including myocardial infarction, coronary artery disease, hypertension, cardiomyopathy, and arrhythmias (1). These heart stressors trigger similar adaptive responses leading to hypertrophy of the remaining cardiomyocytes and activation of cardiac fibroblasts and endothelial cells. Although the physiological purpose of these coordinated cellular events is to repair damaged tissue and preserve cardiac performance, persistent activation of the wound healing process is detrimental and facilitates the progression to heart failure (HF). At the molecular level, HF evolution is characterized by a maladaptive down-regulation of the adult cardiac muscle proteins isoforms such as alpha myosin heavy chain (αMHC) and sarcoplasmic reticulum Ca^2+^-ATPase 2a (Serca2a) and a concomitant up-regulation of the fetal genes such as beta myosin heavy chain (βMHC) and phospholamban (Pln) (2).

The adult-to-fetal gene program switch is paralleled by a reduction of the circulating and cardiac levels of 3,5,3′-triiodothyronine (T3), the main biologically active TH. Such a low T3 state condition (LT3S) is considered an independent factor of poor prognosis both in acute and chronic heart disease and is believed to exert a key role in pathological cardiac remodeling and HF evolution (3–5). In line with these observations, maintenance of TH homeostasis in the setting of cardiovascular diseases (CVD), has been proven to be anti-remodeling and promote cardioprotective effects by affecting several aspects of cardiac physiology including contractility, energy production, myocyte geometry, and fate, as well as angiogenesis and matrix remodeling (6–9).

**Figure 1:**
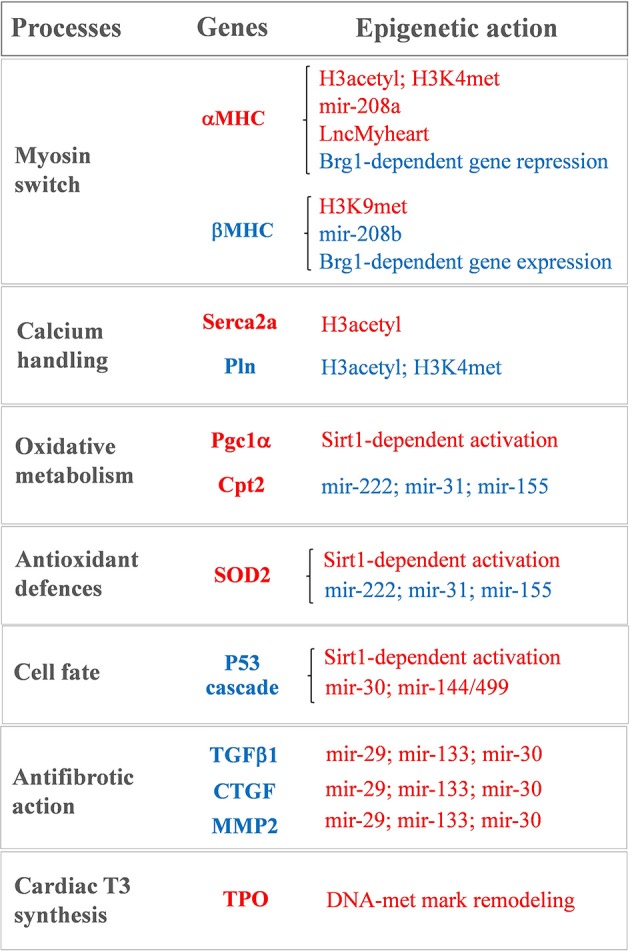
Sketch of the main T3-modulated processes with the corresponding epigenetically regulated genes. In red: genes and epigenetic mechanisms activated by T3. In blue: genes and epigenetic mechanisms inhibited by T3.

It is now clear that THs exert their effects at multiple levels. Increasing evidence points at TH-sensitive epigenetic and post-transcriptional mechanisms as main triggers of the cellular phenotypes that drives cardiac remodeling (see Figure 1). Epigenetic refers to all changes at the nuclear DNA level, that control DNA structure or conformation without affecting its sequence. The most common epigenetic modifications include post-translational histone modifications, DNA methylation, and non-coding RNA transcripts (10–12). Among these mechanisms, hystone modifications and DNA methylations are able to dynamically alter gene expression by modulating chromatin accessibility to transcription factors and coregulators. In particular, opposite alterations of chromatin structure play a crucial role in the switch of cardiac protein isoforms observed during development, in pathological cardiac hypertrophy and in response to altered TH levels (13–18).

TH-sensitive non-coding RNAs are also involved in silencing and activation of gene expression programs relevant to heart physiology and pathophysiology. It is now appreciated that only around 2% of human transcripts are translated into proteins. Many of the remaining non-protein coding transcripts play a pivotal functional role in cardiac tissue homeostasis, which makes them critical targets of therapeutic strategies. The best characterized non-coding RNA species, microRNAs (miRNAs), are 22–23 nucleotide long molecules that turn off gene expression by blocking the translation or inducing the degradation of target transcripts. miRNAs have been involved in a broad range of pathophysiological processes underlying cardiac contractility, cell fate, response to oxidative stress and myocardial fibrosis (19–24). Long non-coding RNAs (lncRNAs), another emerging class of non-coding RNAs, have a length >200 nucleotides. The abundance of lncRNAs in the cardiovascular system is consistent with their crucial role as part of complex regulatory networks governing heart physiology and pathology (25–27). Of particular interest is the recent concept that a cross-talk between lncRNAs and remodelers of chromatin structure may participate in the regulation of cardiac geometry (28, 29).

Increasing experimental evidence suggests that the above described TH-sensitive epigenetic and post-transcriptional mechanisms are critical triggers of maladaptive cardiac remodeling and HF evolution. To reinforce the importance of maintaining TH homeostasis in the context of cardiac injuries, this review is aimed to comprehensively dissect the contribution of epigenetic modifications and non-coding RNAs to the cardioprotective action of T3.

## Chromatin Remodeling in Cardiovascular Disease: an Overview

The function and organization of the genome is dynamically regulated by numerous epigenetic mechanisms that ensure a rapid integration of different signals or inputs (30). Among these mechanisms, post-translational histone modification is crucial to all genome-based activity. Histone proteins are the basic packer and arranger of chromatin structure and can be modified by various post-translational modifications to alter DNA accessibility and gene expression (31). The dynamic transitions between different chromatin conformations rely on a balance between changes that favor a silent state and those that foster a transcriptionally active state (30). In general, histone deacetylation via histone deacetylases (HDACs) allows DNA to wrap around histones more tightly, thus inducing a repressive state. On the contrary, histone acetylation via histone acetyl transferases (HATs) induces chromatin relaxation and gene transcription. Abnormal enrichment of these epigenetic marks move the balance between open and closed chromatin conformations, resulting in an altered transcriptional activity that may favor cardiac disease evolution (32).

For example, an increase of HDAC enzymes conveys the cardiac stress signals to the pathological pro-growth gene program observed in *in viv*o and *in vitro* models of cardiac hypertrophy and adverse remodeling. Along this line, HDAC inhibitors have proved efficacious in several pre-clinical models of HF (33), leading to a reduction in ischemic/reperfusion (IR) injury and post-ischemic remodeling (34–37), hypertrophy (38–43), fibrosis (44, 45), and inflammation (46, 47). On the other hand, abnormal hystone acetylation via p300 HAT has been involved in salt-induced hypertensive HF (48) and agonist-induced cardiac hypertrophy (49).

Histone methylation, another widespread type of chromatin modification, plays important roles in cardiac lineage specification and differentiation (50, 51), as well as in heart development (52, 53), and pathogenesis of congenital and acquired heart diseases (54, 55). In general, histone 3 methylations at lysine 9 (H3K9) and 27 (H3K27) mark regions of transcriptionally inactive heterochromatin, while histone 3 methylations at lysine 4 (H3K4), 36 (H3K36), and 79 (H3K79) are usually associated with transcriptionally active euchromatin (56). A current model suggests that some methylation marks may dynamically change in response to developmental, environmental or cellular stress cues. Methylated histones are then recognized by chromatin regulators that recruit other factors to alter the promoter accessibility (57). Several histone metyltranferase (HMT) and histone demethylase (HDM) have been identified so far that are differentially expressed during cardiomyocytes differentiation and that are involved in the fetal re-programming of CVD (56).

A further important epigenetic mechanism is the methylation of the DNA cytosine residues at the CpG dinucleotide sequences (58). This epigenetic modification is typically associated with gene silencing, although its effect may be dependent on its location with respect to the target gene. DNA methylation is catalyzed by DNA methyltransferases (DNMTs) (59); of the three DNMTs, DNMT1 is responsible for the maintenance of stable methylation patterns, whereas DNMT3a and DNMT3b catalyze *de novo* methylation (59).

Comprehensive studies indicate that alterations in DNA methylation profiles contribute to the orchestration of biological processes involved in CVD. For example, in HF patients altered methylation marks have been found in promoter of genes driving myocyte apoptosis, fibrosis and contractility (60). Targeted DNA methylation profiling of cardiac tissue from patients with dilated cardiomyopathy revealed hypomethylation and significantly elevated gene expression of matrix metalloprotease 2 (MMP2) and connective tissue growth factor (CTGF), two genes important for the turnover and stability of the extracellular matrix (ECM) (61). In human ischemic cardiac disease, a genome-wide DNA methylation analysis integrated with RNA sequencing evidenced a transcriptional reprogramming that repressed oxidative metabolism by promoter hypermethylation of genes involved in mitochondrial respiration, kreb cycle, and fatty acid beta oxidation (62). On the contrary, anaerobic glycolytic genes were found to be hypomethylated, altogether depicting a regression to the fetal gene program of substrate utilization (62).

Overall, the above-described findings indicate that a better understanding of the epigenetic mechanisms underlying the regulation of chromatin scaffold may open a new therapeutic avenue to blunt pathological cardiac growth and progression to HF. At this regard, T3 has been found to play a key role in maintaining the adult cardiac gene expression program via recruitment of chromatin remodeling determinants at TH receptors (THR). Therefore, restoring the physiological TH homeostasis under hypothyroidism or LT3S condition may contrast the pathological cardiac gene re-programming.

## T3-Dependent Regulation of Gene Expression Requires Hystone Modifications

THR transcription factors belong to the steroid hormone receptor super-family and are encoded in two genomic loci that produce two different isoforms (THRA and THRB) (63, 64). The THRA1 and B1 isoforms are widely distributed and exhibit overlapping patterns of expression (64). However, the THRA1 is mainly expressed in skeletal and cardiac muscle while the THRB1 isoform is more abundant in liver, kidney, brain, and thyroid (63, 64). The use of knock-in animal models harboring THR mutations has provided helpful insight to deciphering the specific role of THRA and B isoforms and distinguishing between canonical and non-canonical cardio-metabolic action of T3. A complete description of this literature is beyond the scope of this review the interested readers are referred to recent papers (65, 66). Briefly, THRA plays a crucial role in post-natal development and cardiac function, whereas THRB is mainly involved in controlling liver metabolism, the hypophysis pituitary thyroid axis, the circulating levels of TH, and the development of the retina and the inner ear (65). In addition, mice expressing mutant THRs that cannot bind DNA provided *in vivo* evidence that relevant physiological effects mediated by THRs are independent from DNA binding and direct activation of gene expression. These so-called non-canonical TH signaling includes energy metabolism, locomotor activity, and heart rate (66).

When acting through the canonical pathways, THR form homodimers or heterodimers with retinoid X receptor and bind to specific THR response element (TRE) in the regulatory region of the target genes. In contrast to other receptors of the family, unliganded THR localize to nucleus and interact with nucleosome-embedded DNA (67, 68). It has long been recognized that THR are master regulators of the chromosomal structure in driving transcriptional activation or repression (69). THR regulation of gene expression is commonly described by a model of chromatin folding switch (63). On T3-positively regulated target genes, unliganded, chromatin-bound THRs interact with transcriptional co-repressors such as Silencing Mediator of Retinoic acid receptor and TH receptor (SMRT) and Nuclear Corepressor- 1 (NCoR1) and represses transcription through HDACs recruitment (70–74). Binding of the hormone is supposed to induce a conformational change of THR, leading to detachment of corepressors and binding of coactivators such as Steroid Receptor Coactivator 1 (Src1) or TH Receptor-Associated Protein (TRAP) (75–78). In turn, coactivators recruit p300 HAT and form an open status of chromatin that allows the binding of polymerase II transcriptional machinery and the activation of target gene expression (63, 79–89).

This mechanism of action has important physiological implications in presence of TH dyshomeostasis. At low TH concentrations, as in the presence of hypothyroidism, or low T3 state, the unliganded THR is expected to suppress transcription instead of operating as an inactive, passive receptor. Consistently, hypothyroidism models present a much more sever phenotype than THRA and THRB double knockout mouse model (81–84).

The mechanism of transcriptional regulation at negatively regulated THR targets, that are repressed by T3 binding and activated by unbound THR, is less understood (85). It has been proposed that the corepressors and coactivators act in an opposite manner on this kind of target genes, but the exact underlying molecular mechanism is still unclear (85, 86). Genome-wide profiling of THR binding sites in experimental model of hypo- and hyperthyroidism highlighted additional mechanisms of positive vs. negative regulation of gene expression by T3 (63, 87). These studies demonstrated that: (1) a considerable number of intronic and intergenic regions exists where THR are dynamically recruited upon T3 binding to activate transcription; (2) different DNA binding motifs are preferentially enriched at T3 activated or repressed target sites; (3) T3-facilitated gene repression is associated to reduced chromatin accessibility or decreased THR occupancy (63, 87). In the latter case, transcriptional down-regulation could be mediated by reduced affinity of the T3-liganded THR for specific DNA binding motifs (87).

Notwithstanding being recently revisited and implemented (63, 87), the bimodal switch model of THR-mediated chromatin remodeling still holds true for the antithetical regulation of the myocardial MHC, Serca2a, and phospholamban gene expression observed during cardiac development and disease (15–18, 88–90) (see Figure 1). A role of differential histone acetylation in T3 regulation of myocardial αMHC gene expression was first demonstrated in a hypothyroid rat model (15). In that study, treatment with the HDAC inhibitor trichostatin A (TSA) was unable to switch the MHC expression in the absence of T3. However, TSA potentiated the activation of the αMHC gene when administered along with T3, supporting the hypothesis that an altered histone acetylation is involved in the T3-mediated regulation of MHC gene transcription. Chromatin immunoprecipitation subsequent studies confirmed that TH levels induced specific histone modifications markers at the α and βMHC promoter locus with opposite effect on MHC isoform expression (16–18) (see Figure 1). In particular hypothyroidism determined a reduction of the H3 acetylation and H3K4me marks at αMHC promoter, along with an increase of H3K9met at the same locus. Also, hypothyroidism enhanced βMHC expression by increasing H3 acetylation and H3K4me at the βMHC locus. Reversal of the hypothyroid condition restored the marks of active chromatin on αMHC while repressing them at the βMHC promoter (16–18) (see Figure 1). Interestingly fetal or pathologically-stressed heart showed similar epigenetic marks at the repressed αMHC promoter as observed under hypothyroidism (14). Such outcome requires the activity of the ATP-dependent chromatin remodeler Brahma-Related Gene 1 (Brg1). Brg1 is known to govern two independent pathways that drive cardiac growth and differentiation during development and in pathological condition. In the fetal life a high level of Brg1 maintains myocytes in an embryonic state, with inhibition of αMHC expression and activation of βMHC (12). During cardiomyocyte differentiation Brg1 expression is significantly reduced, facilitating the switch to αMHC expression (12). A re-activation of Brg1 is observed in the adult heart of cardiac patients or experimentally stressed animal models, and drives the hypothyroid-like epigenetic events leading to the pathological α to βMHC switch (14). The opposite effects of T3 and Brg1 in the epigenetic control of MHC isoform expression, along with the different myocardial level of T3 in the fetal and adult heart, suggest an intriguing novel mechanism whereby T3 may affect αMHC myocardial content via antagonizing the Brg1 activity (see Figure 1).

Epigenetic modification is also required for the T3-dependent activation of Serca2a, another important player of myocardial contractility (88) (see Figure 1). Serca2a is a critical determinant of the Ca^2+^ uptake within the sarcoplasmic reticulum (SR) thus affecting the excitation–contraction coupling. Reduced expression of Serca2a has been widely reported in both systolic and diastolic HF (91–93) while Serca2a overexpression was documented to ameliorate cardiac performance and to reduce the incidence of ventricular tachyarrhythmias (94–96). Therefore, enhanced Serca2a expression is considered a potential master plan to blunt cardiac dysfunction and arrhythmias. However, efficacious pharmacological interventions to restore Serca2a levels are not available, and adenoviral-mediated gene delivery of Serca2a in HF patients failed to rescue the reduced ejection fraction (97). As for αMHC, Serca2a expression at mRNA and protein levels increases after birth in parallel to a surge of T3 level. The decreased expression of Serca2a observed in hypothyroidism can be normalized by restoring the hormonal levels through TH administration (98). In an *ex vivo* study on human myocardial biopsies, T3 replacement at physiological doses rescued the intracellular flux of Ca^2+^ that was compromised following long-term T3 deprivation (99). Thus, it is plausible that the LT3S, observed during cardiac disease evolution, may promote Serca2a repression. In line with this interpretation, at Serca2a promoter the liganded THR recruit p300 HAT and TRAP regulators in sequential steps to induce hystone acetylation and increase protein synthesis (88).

Serca2a activity is reversibly inhibited by the phosphoprotein Pln, thus modulators of the Serca2a/Pln regulatome are important determinant of Ca^2+^ cycling kinetics and cardiac contractility (100). It is clearly established that T3 is able to suppress the Pln gene expression in the heart (101–104). There is a tight correspondence between the entity of Ca^2+^ uptake within the SR and the Pln to Serca2a ratio in hypothyroidism, euthyroidism, and hyperthyroidism conditions, which determines the cardiac performance (103). The precise mechanism of T3 action on Pln remained unclear until the study of Belakavadi and coworkers (90). These authors revealed that T3-dependent down-regulation of Pln in cardiac myocytes is mediated by THRA1 and involves the hormone-directed recruitment of HDAC and HDM activities to decrease both histone H3 acetylation and H3K4met epigenetic marks (90) (see Figure 1).

## T3 and DNA Methylation

In contrast to histone modifications, the effect of TH on DNA methylation is less well-studied. The available data are limited to extra cardiac tissues and suggest the involvement of TH in the regulation of DNA methylating enzymes. Persistent exposure of neonatal rats to antithyroid drug propylthiouracil leads to considerable changes of DNMT expression with increased induction of oxidative stress and up-regulation of p53 in adult liver (105, 106). Newly diagnosed hyperthyroid patients had genome-wide hypomethylation and lower DNMT1 expression in T and B lymphocytes; while relief of hyperthyroidism with antithyroid drugs restored the global DNA methylation and DNMT1 expression (107). These findings raise the intriguing working hypothesis that differential DNA methylation in circulating blood cells could be exploited to stratify patients according to their thyroid state. If validated by dedicated studies, such an approach could be particularly useful to guide better personalized treatment even in patients with milder alteration of thyroid homeostasis such as LT3S.

The role of TH in the direct modulation of DNMT expression has been extensively analyzed in processes that require global changes of tissue architecture such as Xenopus metamorphosis and post-natal neurological development. A recent study showed that the critical gene for *de novo* DNA methylation, DNMT3a, is controlled by T3 in developing frog tissues. The expression of DNMT3a increased as endogenous plasma T3 rises, and could be induced by exogenous T3 administration (108). The same authors also found that the post-natal surge of T3 levels in mouse brain was paralleled by increased expression of DNMT3a and that the treatment of mouse neuronal cells with T3 caused rapid induction of DNMT3a mRNA (109). Based on these findings it has been proposed that T3 modulation of DNMT3a may represent an evolutionarily conserved mechanism for regulating the post-natal brain differentiation by inducing genome-wide changes in DNA methylation marks. As well as the brain, the heart is a terminally differentiated organ whose post-natal maturation requires a surge of T3 circulating level. Therefore, it can be speculated that similar T3-mediated epigenetic DNA modification as in the brain may be at work to determine heart differentiation. Along this line, it is also conceivable that the LT3S state observed in CVD evolution may favor altered DNA methylation underlying the regression to the fetal gene program. Unfortunately, whether T3-dependent DNA methylation plays a role in cardiac pathophysiology remains an open question that deserves further investigations.

There is, however, emerging evidence indicating that altered DNA methylation may be involved in the setting of myocardial LT3S in patients with CVD. By using RNA-sequencing, it was recently demonstrated that the human myocardium expresses the machinery for TH biosynthesis (110, 111). In patients with ischemic cardiomyopathy, the mRNA levels of thyroperoxidase (TPO), a key component of this TH enzymatic machinery, was reduced in association with reduced T3 levels and altered methylation pattern of the TPO gene (see Figure 1). Also, retrieval of data from methylation analyses in vessel from atheroschlerotic patients confirmed similar methylation pattern in the same CpG sites, thus suggesting that altered methylation marks at TPO gene may contribute to the reduced local production of T3 observed in CVD (110).

Finally, an indirect route by which T3 might regulate cardiac DNA methylation, is via interaction with the sympathetic nervous system (SNS). It is well-established that T3 can alter the responsiveness to sympathetic stimulation by enhancing the function and density of the adrenergic receptors within the cardiovascular system. The available data also indicate that activation of adrenergic signaling modulates epigenetic mechanisms of DNA methylation implicated in cardiac hypertrophy and HF (112, 113). These intriguing premises lay the ground for future studies aimed at elucidating the cross-talk between T3 and the SNS in the modulation of cardiac DNA methylation in physiological and pathological conditions.

## T3 Regulation of Cardiac microRNAs

Regulation of myocardial miRNA is an emerging mechanism for T_3_-mediated epigenetic control of cardiac gene expression. As single miRNAs can target several genes within functional related biological processes, controlling the expression of one microRNA can affect whole gene networks and alter the phenotype of complex diseases (20). Accordingly, T3-regulated miRNAs have been shown to counteract many noxious processes underlying adverse cardiac remodeling.

A recent miRNA profiling in hearts of hypothyroid and hyperthyroid mice revealed a signature of T3-responsive miRNAs, including miR-208a, that are predicted to repress the transduction of the prohypertrophic, pathological cascade (114). miRNA-208a is one of the main heart-enriched miRNA that plays a key role in cardiac physiopathology, it belongs to a miRNA family that also comprises miR-208b and its promoter is located in an intronic region of the αMHC gene. Within the heart, miR-208a and miR-208b are implicated in the MHC isoform switch during heart development and disease. In mice hearts physiological levels of miR-208a are necessary for a correct cardiac electrical activity (115). Conversely, cardiac overexpression of miR-208a causes pathological cardiac hypertrophy by targeting TRAP1 and myostatin, that are negative regulators of muscle growth (115). Reduction of myocardial TH levels in cardiac disorders favors αMHC and miR-208a repression while administration of THs to cardiomyocytes in culture significantly upregulates the αMHC/miR-208a expression-ratio and decreases the βMHC/miR-208b expression-ratio (115) (see Figure 1). These data add a further level of complexity to the T3-dependent regulation of MHC expression and suggests that physiological TH concentrations are required to ensure proper miRNA levels and to preserve MHC composition.

In experimental models of post-ischemic LT3S, T3 replacement was paralleled by rescued levels of miR-30a expression within the area at risk (116). The miR-30 family members are expressed at high levels in the adult heart, and are significantly down-regulated in human HF, in experimental IR, and *in vitro* after oxidative stress (116–118). miR-30 has been shown to target p53, a well-known activator of mitochondrial apoptosis and necrotic pathways (118–120). Consistently, T3 replacement following cardiac IR was associated to a down-regulation of the p53 signaling along with better preserved mitochondrial function and decreased cell death (116). Also, T3 physiological replacement in hypoxia-stressed neonatal rat cardiomyocytes in culture prevented both miR-30a down-regulation and p53 activation. The protective action of T3 was greatly inhibited by miR-30a knockdown. Collectively, these findings support a novel T3-dependent cardioprotective mechanism that influences mitochondrial function and is driven by the miR-30a/p53 axis (116) (see Figure 1).

T3 is also involved in the regulation antifibrotic process following cardiac IR stress via up-regulation of miR-29, -30c, and -133 (121). A decrease of these miRNAs following myocardial damage leads to fibrosis and conduction alterations via up-regulation of an array of proteins involved in ECM remodeling (21, 24, 122–124). It has been shown that a LT3S in the early phase of the post-IR setting represents a permissive condition for a long term maintenance of high levels of transforming growth factor beta 1 (TGFβ1) (121). In turn, raised TGFβ1 levels inhibited the antifibrotic miR-29c, miR-30c, and miR-133a and de-repressed their profibrotic targets MMP2 and CTGF. Ultimately, these early molecular events led to adverse remodeling and reduced cardiac contractility (117). LT3S correction through timely T3 infusion hampered the inhibitory effect of a protracted TGFβ1 up-regulation on the antifibrotic miRNA pathway, thus decreasing fibrotic tissue deposition, scar size, and heart dysfunction (121) (see Figure 1).

A recent comprehensive miRNA profiling provided novel indication that an early post-IR T3 treatment associates to the modulation of a host of myocardial miRNAs critically implicated in the orchestration of heart development, function, and disease (122). Among the newly identified miRNAs that are up-regulated by T3, the miR-144/451 cluster and miR-499 have documented protective effects in the infarcted myocardium by limiting cardiomyocyte death and excessive mitochondrial fission (125, 126). On the other hand, miR-31, -155, and -222, that are down-regulated by T3, have been shown to promote cardiac dysfunction and adverse remodeling in both HF and ischemic heart disease (127–130). In accordance, the computational integration of the T3 differentially expressed mRNAs and T3 differentially expressed miRNAs identified a network of T3-modulated regulatory circuitries (122). Such connections are predicted not only to inhibit the harmful signaling cascade leading to cardiomyocyte loss, mitochondrial alterations, inflammation and extracellular matrix remodeling, but also to potentiate protective pathways that preserve mitochondrial quality control and oxidative metabolism including mitochondrial fusion, protein import and folding, mitochondrial antioxidant activity, and carnitine shuttle (122) (see Figure 1). Such data are in agreement with the notion that T3 plays a pivotal role in cellular growth, homeostasis and metabolism (131, 132).

The available evidence on experimental models indicates that the protective cardiac action of T3 is under the synergistic control of T3-responsive miRNAs. Also, a protracted post-ischemic LT3S seems to be a permissive condition for the maintenance of the fetal miRNA program. Such a fetal gene recapitulation contributes to the pathological changes associated with progressive cardiac dysfunction (117, 133). Indeed, in pathological hypertrophy, a striking analogy has been found between the miRNA expression profile found in human HF and that observed in the hearts of 12–14 weeks old fetuses (23). Therefore, based on the published data, a model can be envisioned in which stress stimuli alter the intra-cardiac TH signaling leading to reduced T3 level. In this context, maintenance of T3 cardiac homeostasis through T3 replacement might blunt the dysregulation of T3-dependent miRNAs and their target mRNAs thus limiting adverse remodeling.

## T3 Regulation of Cardiac Long Non-coding RNA

LncRNAs, another class of non-protein coding DNA products, have been shown to play a major role in many aspects of myocardial gene regulation, especially the epigenetic processes that underpin organogenesis and remodeling of cardiac architecture (134). In particular, lncRNAs have been proposed to bind certain methyltransferases and demethylases and to guide them to specific genomic regulatory region or to inhibit their interaction with the chromating scaffold and DNA (134). Among the various species of lncRNA, the natural antisense transcripts (NATs) form an abundant class of regulatory molecules that are transcribed by the opposite strand of protein coding genes. One of them is located within the 4.5-kb intergenic space between the αMHC and βMHC genes and is implicated in the regulation of the MHC switch (135). Within this intergenic region a bifunctional promoter has been found to coordinately control the transcription of the αMHC sense mRNA and βMHC antisense RNA (136). The βMHC NAT extend over the βMHC gene and its promoter. Stimulation of such intergenic transcription has been supposed to silence βMHC sense transcription via RNA-RNA interference to favor the αMHC isoform expression (135, 136). Compelling evidence in hypothyroid and hyperthyroid models indicate that T3 is a positive regulator of this NAT within the myocardial tissue (16, 137). It has been suggested that the T3-dependent NAT binds chromatin repressing complexes such as HDAC and HMT to alter chromatin accessibility at the βMHC promoter (16). According to this model, in pathological condition, reduced T3 levels repress the intergenic NAT along with αMHC gene transcription while relieving the repression at βMHC gene, which allows a rapid α-to βMHC switch (16).

Recently a lncRNA, named Myheart (Mhrt), located in the antisense region between the αMHC and βMHC has been found to protect the heart against pathological hypertrophy (28). This action seems not to involve RNA–RNA sequence interference between Mhrt and the βMHC transcript as previously supposed. Instead, Mhrt binds to and represses the activity of Brg1 protein, the chromatin remodeler that triggers the fetal gene reprogramming of cardiac myopathy (28). Following pathologic cardiac stress, the Brg1-HDAC chromatin repressor complex is up-regulated leading to the inhibition of Mhrt transcription and development of cardiomyopathy, which is prevented by reestablishing the pre stress levels of Mhrt (28).

## Indirect Transcriptional Regulation by THs via Sirtuin Mediated Epigenetic Mechanisms

Sirtuins (Sirt) are a family of NAD^+^-dependent deacetylating enzymes. Sirt1, the best characterized one, coordinates the metabolic adaptation of the whole organism by modulating the transcription pattern in response to the cell need (138). Several epigenetic mechanisms have been so far identified for Sirt1-mediated regulation of gene expression. Sirt1 can lead to transcriptional repression through direct histone deacetylation, or via methylation of histones and DNA (138). Secondly, Sirt1 can bind and deacetylate a wide range of transcription regulators including TR, thus affecting the expression of target genes positively or negatively (138). Also, Sirt1 deacetylates histone-modifying enzymes to regulate their function. In this manner, Sirt1 interacts with p300 HAT and inhibits its enzymatic activity to promote nucleosomal histones hypoacetylation and alter gene expression outcomes (139).

Sirt1 plays a critical role in CVD physiopathology being involved in the regulation of several cellular metabolic pathways as well as in the death or survival decision making (140). For this reason, Sirt1 is the most extensively studied sirtuin in the cardiovascular system (140). The available literature indicates that Sirt1 can exert beneficial or noxious cardiac effects in a dose dependent manner (140–142). In mouse models of cardiac IR a mild to moderate Sirt1 overexpression (up to about sevenfold) proved cardioprotective by decreasing the pro-apoptotic cascade and increasing the mitochondrial antioxidant defenses (141). On the contrary, excessive Sirt1 up-regulation (more than 10 fold) elicits opposite effects including mitochondrial dysfunctions and low energy production associated to decreased expression of the peroxisome proliferator activated receptor gamma coactivator 1 alpha (PGC-1α) (142). These data indicate that to afford cardioprotection the levels of Sirt1 must be rigorously controlled (140).

TH state has been shown to affect Sirt1 activity either directly, by affecting the protein levels, and indirectly, by influencing the cytosolic concentration of NAD^+^ that is generated through oxidative phosphorylation (143, 144). Also, Sirt1 is required to promote THR-mediated gene expression. In liver tissue, Sirt1 and THRB1 interact with each other to enhance T3-responsiveness (145, 146). Accordingly, T3 has been reported to share many metabolic mechanisms with Sirt1 (147, 148) (see Figure 1). Even if the T3-Sirt1 connection has been extensively characterized only in extra cardiac tissue, we recently found that T3 regulates the same antioxidant, antifibrotic and prosurvival cardioprotective mechanisms as Sirt1 (122, 142, 149–151), thus suggesting that the T3-Sirt1 cross-talk may be a generalized mechanism to increase the panel of T3-regulated processes under different patophysiolgical conditions. Finally, it is worth mentioning the emerging cardio-metabolic action of 3,5 diiodothyronine (T2) via Sirt1 deacetylation pathways (152, 153). T2 is an endogenous active metabolite of T3 that is able to modulate similar processes as T3 in many tissues without manifesting all the deleterious cardiac effect of hyperthyroidism, even when used at pharmacological doses (152, 153). One proposed mechanism of action is the rapid activation of Sirt1 that deacetylates Pgc-1α and Sterol regulatory element-binding proteins-1c (Srepb-1c). These transcription factors then trigger a cascade of events improving mitochondrial biogenesis and energy metabolism, while preventing fat accumulation and diet induced insulin resistance (153). These promising findings encourage further studies to directly explore the therapeutic use of T2 under cardiac disease conditions.

## Future Perspectives and Concluding Remarks

Overall the available data on T3 cardiac effects should provide helpful guidance to manage hypothyroidism or LT3S conditions optimizing the benefit to risk profile.

Although T3 mediates the vast majority of physiological processes (see Figure 1), LT4 is still considered the unique choice to treat hypothyroidism. Unfortunately, due to altered peripheral conversion of T3 from T4, ~10–20% of patients taking T4 monotherapy experience symptom persistence or recurrence despite normal TH test (154–158). This outcome highlights the need to consider better personalized replacement approaches for a certain patient population. Individualized T3 or combination therapies should be developed to mimic a more physiological intratissutal thyroid hormone signaling. At this regard, the dynamic nature of some epigenetic modifications that are particularly sensitive to altered TH homeostasis, should be exploited by the biomarker discovery research to distinguish patients that may best benefit from T4 monotherapy or combined replacement, thus avoiding the potential adverse effect of thyrotoxicosis.

As a second key point, it has been a long standing dogma that the LT3S in the hyperacute post-IR phase may be beneficial by reducing energy consumption (159). However, increasing clinical and experimental data indicate that the persistence of a LT3S favors cardiac disease evolution and worsens patient prognosis (6, 160, 161). Importantly, to be efficacious against adverse remodeling, the timing of LT3S correction should take into account the regenerative window that is lost in the late post-IR phase (162). Accordingly, T3 administration started 1 week after MI ameliorated heart performance without reversing cardiac remodeling (163). By contrast, THs administered early after IR enhanced cardiac function and prevented left ventricle remodeling (116, 121, 122, 164, 165). These finding are in line with the key role of T3 as main modulator of cardiac repair/regeneration, a process that is critically affected by dynamic epigenetic mechanisms. As for other cardiac epigenetic regulators, such as plant compounds or selected exosomes (166, 167), lower T3 doses proved more efficacious than pharmacological treatments in contrasting the noxious pathways of adverse remodeling (168, 169). Such information on optimized dose and timing for T3 delivery after IR should provide clinically translatable protocol based on inexpensive, commercially available T3.

## Author Contributions

GI and FF conceived the manuscript. FF has written the manuscript with input from all authors. GN and LP contributed analysis of the bibliographic material. All authors have provided critical discussion and have revised the final manuscript.

### Conflict of Interest Statement

The authors declare that the research was conducted in the absence of any commercial or financial relationships that could be construed as a potential conflict of interest.
